# Cytokine profiles and their correlation with clinical and blood parameters in rheumatoid arthritis and systemic lupus erythematosus

**DOI:** 10.1038/s41598-024-72564-z

**Published:** 2024-10-08

**Authors:** Nancy Paola Duarte-Delgado, Katherine Segura, Oscar Gómez, Sandra Pulido, Catherin Tovar-Sánchez, J. M. Bello-Gualtero, Daniel G. Fernández-Ávila, Sandra B. Amado-Garzón, Consuelo Romero-Sanchez, Stefano Cacciatore, Luz-Stella Rodríguez C.

**Affiliations:** 1 Facultad de Medicina, Instituto de Genética Humana, Pontificia Universidad Javeriana, Carrera 7 # 40-62, Bogotá, Colombia; 2 Departamento de Psiquiatría, Hospital Universitario San Ignacio, Carrera 7 # 40-62, Bogotá, Colombia; 3 Grupo de Inmunología Clínica Aplicada, Servicio de Reumatología - Hospital Militar Central, Facultad de Medicina – Universidad Militar Nueva Granada, Tv. 3C No. 49 – 02, Bogotá, Colombia; 4 Unidad de Reumatología, Hospital Universitario San Ignacio, Carrera 7 # 40-62, Bogotá, Colombia; 5 Departamento de Medicina Interna, Pontificia Universidad Javeriana, Carrera 7 # 40-62, Bogotá, Colombia; 6 Bioinformatics Unit, International Centre for Genetic Engineering and Biotechnology (ICGEB), Cape Town, 7925 South Africa

**Keywords:** Cytokines, Rheumatoid arthritis, Systemic lupus erythematosus, Spearman’s correlation, Autoimmunity, Cytokines

## Abstract

The abnormal biological activity of cytokines and their imbalance are implicated in developing rheumatoid arthritis (RA) and systemic lupus erythematosus (SLE). Cytokine levels were measured in RA and SLE patients and compared to healthy controls using the Wilcoxon rank sum test and Kruskal–Wallis test. The relationship between cytokine levels and blood and clinical parameters was assessed using Spearman's correlation test. Compared to healthy controls, both RA and SLE patients exhibited elevated levels of GM-CSF, CX3CL1, IFN-α2, IL-12p70, IL-17A, TNF-α, IL-1β, and IFN-γ, which is evidence of their shared inflammatory signature. IL-2 levels were elevated exclusively in RA patients, while MCP-1 and IL-10 were uniquely increased in SLE patients. Notably, TNF-α showed the most significant increase in SLE patients. IL-4 was elevated in SLE patients with nephritis, correlating with IL-6, IL-10, sCD40L, and IL-8, suggesting B cell involvement in lupus nephritis. The negative correlation between CX3CL1 and TNF-α with HDL in RA and SLE respectively, highlights the potential association of these inflammatory markers with cardiovascular risk. These findings underscore the complex cytokine interplay in RA and SLE. CX3CL1 emerges as a potential therapeutic target for RA, while TNF-α and IL-4 show promise as therapeutic targets for SLE.

## Introduction

Systemic lupus erythematosus (SLE) and rheumatoid arthritis (RA) are systemic and heterogeneous autoimmune diseases with a worldwide distribution that affect mainly women^1^. In RA and SLE the exacerbated activation of the immune system leads to the loss of immune tolerance to autoantigens, the presence of autoantibodies and the hyperactivity of multiple immune cells^1^. RA is primarily a joint disease associated with autoantibodies targeting modified self-epitopes^2^, while SLE is recognized by its diverse organ involvement and the presence of autoantibodies directed against self-nuclear antigens^3^. RA prevalence in Colombia is 240 per 100.000 inhabitants^4^ and for SLE is 91.9 per 100.000 inhabitants^5^.

The greatest genetic risk for the development of autoimmune diseases including RA and SLE, has been found at the major histocompatibility complex (MHC) locus but there are non-MHC genes like CTLA4, PTPN22 and TNF that have also been associated with this risk^6^. Environmental influences like infections, xenobiotics, food and its effects on the microbiome, air pollution and personal lifestyles also play a crucial role in the development of these diseases^7^. Nevertheless, it is the interaction between these genetic and environmental factors responsible for inducing disease evolution from the genetic risk factor stage to subclinical autoimmunity, to early clinical signs and symptoms that result in a disease phenotype matching classification or diagnostic criteria^7^. Their similar pathophysiology, as well as the common genetic and environmental factors that trigger them, point to a common origin of these diseases in what is known as the autoimmune tautology^8^.

Cytokine-mediated pathways are important in the development of RA and SLE. Both are characterized by a high production of cytokines with mainly proinflammatory functions and the deregulation of anti-inflammatory cytokines, which promotes the induction of autoimmunity by activating signaling pathways related to chronic inflammation, autoantibody overproduction and end-organ effects^9,10^. Nevertheless, there is still controversy regarding the contribution of specific cytokines in the pathogenesis of these diseases^11^.

Immune phenotypes, including cytokine levels, vary according to ethnicity due to the influence of the exposure to endemic pathogens and the specific genetic make-up, so cytokines like IFN-α2 and TNF-α were increased in healthy African women compared to European women^12^. These differences in cytokine levels according to ethnicity have also been proven in SLE patients, where African patients have increased levels of TNF-α, IL-13, and IL-4 compared to European^13^. The Colombian population is particularly interesting due to its high levels of ethnic admixture between African, Native American and European ancestral populations^14^. Population admixture may result in a unique cytokine profile that might be important to characterize to propose specific cytokine biomarkers and targets for this Latin American population. This exploratory study aimed to evaluate the plasma cytokine profile in a group of Colombian women with RA and SLE.

## Methods

### Participants’ recruitment

A total of 24 women with SLE and 24 women with RA were recruited at Hospital Universitario San Ignacio and the Hospital Militar Central (Bogotá D.C., Colombia). The women aged 19 to 55 years had a body mass index (BMI) between 18.5 and less than 30. Both groups of patients had a disease duration of at least 2 years. Patients with RA met the classification criteria according to ACR/EULAR 2010^15^ and the disease activity evaluated by the Disease Activity Score 28- Erythrocyte Sedimentation Rate (DAS28-ESR)^16^ ranged between 3.2 and 5.2. The SLE patients met the Systemic Lupus International Collaborating Clinics classification criteria^17^, and the disease activity evaluated by the SLEDAI (Systemic Lupus Erythematosus Disease Activity Index)^18^ ranged between 4 and 12. Women who were excluded were smokers, obese, pregnant, had a concomitant systemic disease (e.g., diabetes and cancer), or had autoimmune diseases different from RA and SLE including antiphospholipid syndrome and scleroderma. We also excluded SLE women with neuropsychiatric symptoms and/or lupus nephritis stage IV or V, and SLE and RA patients who had received biologic treatment in the last 3 months or rituximab in the last 12 months, or that had received antibiotic treatment in the last 3 months. Healthy controls (HCs) were selected by matching their age and BMI to that of RA and SLE patients. Informed consent was obtained from participants. This project was approved by the Research and Ethics Committees of the Hospital Universitario San Ignacio and the Hospital Militar Central and is governed by the ethical principles for research in human beings included in the Declaration of Helsinki.

### Plasma sample collection

All fasting participants had 15 mL of peripheral blood collected in EDTA tubes. Plasma was isolated by centrifugation at 3000 rpm (Allegra X centrifuge) and subsequently stored at—80 °C until cytokine measurement. Plasma was chosen as the biological matrix for cytokine analysis due to its superior stability and accuracy compared to serum. The clotting process involved in serum collection can lead to platelet activation and cytokine release, introducing variability and compromising data integrity. Plasma, collected with anticoagulants, prevents these issues, ensuring more reliable cytokine measurements^19^.

### Laboratory tests

All patients and controls underwent complete blood count analysis including ESR (Erythrocyte sedimentation rate). Lipid profile parameters (High-density lipoprotein (HDL), low-density lipoprotein (LDL), and total cholesterol), glycemia, glycosylated hemoglobin (HbA1c), and C-reactive protein (CRP) were also evaluated. Only patients with SLE were evaluated for serum anti-dsDNA antibodies and C3 and C4 protein levels.

### Quantification of cytokines

The Milliplex® Map human cytokine/chemokine magnetic bead-based panel (Merck Millipore, Germany) was used according to the manufacturer's instructions to evaluate the plasma levels of 17 cytokines: Granulocyte–macrophage colony-stimulating factor (GM-CSF), CX3CL1, interferon-alpha 2 (IFN-α2), interferon-gamma (IFN- γ), interleukin – 10 (IL-10), macrophage-derived chemokine (MDC), interleukin-12 (IL-12p70), soluble CD40 ligand (sCD40L), interleukin-17 (IL-17A), interleukin-1 beta (IL-1β), interleukin-2 (IL-2), interleukin-4 (IL-4), interleukin-6 (IL-6), interleukin-8 (IL-8), monocyte chemoattractant protein-1 (MCP-1), tumor necrosis alpha (TNF-α) and vascular endothelial growth factor (VEGF). We used the readout platform Luminex® xMAP® and analyzed the data using Milliplex Analyst software version 5.1 (https://www.merckmillipore.com/) to calculate the cytokine concentration.

### Statistical analysis

Statistical analysis and plots were created using R software version 4.3.1 (https://cran.r-project.org/). For the numerical covariates (e.g., age and cytokine concentration) the median and interquartile range (IQR) were reported. Wilcoxon rank sum test and Kruskal–Wallis test were used to establish statistically significant differences between the study groups. Categorical variables were expressed as numbers and percentages, and the *p*-values were calculated with Fisher’s exact test. These calculations were done using the R package “KODAMA”^20^.

For cytokine values below the lower detection limit, imputation to half of the minimum detectable concentration was performed. The multiple comparisons between the groups (RA vs HC, SLE vs HC, and RA vs SLE) were evaluated through Dunn’s test using the R package “FSA”^21^. The *p*-values were adjusted with the Bonferroni method and were considered statistically significant if they were less than 0.05. Then, cytokine levels were plotted in a heatmap using the R package “pheatmap”^22^. To evaluate the performance of the cytokine levels in the classification of RA and SLE patients, the Receiver operating characteristic (ROC) curve analysis was performed with the R package “pROC”^23^.

Spearman’s test was used to calculate the correlation coefficient (rho) of the cytokine levels with blood and clinical parameters. Correlation values of *p* < 0.05 were considered statistically significant. For the network analysis of the correlations, a distance matrix between the variables was set up and then the Floyd-Marshal algorithm was used to calculate the shortest paths in the distance matrix. Multidimensional scaling (MDS) was employed for dimensionality reduction and then the output of the KODAMA algorithm^24^ was visualized in a two-dimensional space as a network of variables.

## Results

### Characteristics of the participants

We profiled the cytokine levels of 77 Colombian women, including 24 SLE patients, 24 RA patients, and 29 age- and BMI-matched HCs. The clinical and demographic characteristics of patients with RA, SLE, and HCs are summarized in Table 1. The median ages of patients with RA, SLE, and HCs were 43, 37, and 40 years, respectively. The median disease duration was higher in SLE (5.8 years) than in RA (4.76 years), but the difference was not statistically significant. The disease activity scores showed a median of 3.55 for the DAS28-ESR and 6 for the SLEDAI. There were statistically significant differences between the study groups in the numbers of lymphocytes and neutrophils and the HDL, LDL, and total cholesterol concentrations. Lymphocytes were lower in RA and SLE patients compared to HCs. Neutrophils were higher in RA and SLE patients compared to HCs. HDL, LDL, and total cholesterol were lower in RA and SLE patients compared to HCs. RA patients were mainly prescribed methotrexate (66.7%) and oral glucocorticoids (70.8%), while SLE patients predominantly took antimalarial drugs (hydroxychloroquine and chloroquine) (91.7%) and oral glucocorticoids (50%) (Table S1).

**Table 1:** Clinical and demographic characteristics of rheumatoid arthritis (RA), systemic lupus erythematosus (SLE) patients and healthy controls (HC).

Feature	HC (n=29)	RA (n=24)	SLE (n=24)	*p*-value
Age, median [IQR]	40 [31–50]	43 [40.2–49]	37 [27–42.5]	0.068
BMI, median [IQR]	24.5 [22.2–26.7]	24.8 [23.2–26.6]	25.1 [23.4–26.9]	0.764
Familiar antecedent of autoimmunity (%)	0 (0.0)	10 (41.7)	5 (20.8)	**0.0002**
Disease duration, median [IQR]		4.7 [3.0–8.5]	5.8 [3.5–12.2]	0.222
SLEDAI score, median [IQR]			6 [ 4–6]	
DAS28 ESR score, median [IQR]		3.55 [ 3.4–3.8]		
Renal involvement in SLE (%)			10 (41.7)	
HDL, median [IQR]	51.5 [44–58.7]	48.5 [38.2–57.2]	45.5 [38.5–50]	**0.040**
LDL, median [IQR]	123 [97.9–147]	110 [86.7–122]	91.5 [72.9–113]	**0.014**
Total cholesterol, median [IQR]	202 [178–240]	176 [156–215]	164 [144–188]	**0.006**
Glucose, median [IQR]	86 [83–91.7]	83 [76.7–92.1]	83.5 [76–92.2]	0.286
HbA1c, median [IQR]	5.2 [5–5.4]	5.2 [5–5.3]	5.2 [5–5.5]	0.864
Hematocrit, median [IQR]	44.1 [41.8–44.8]	40.6 [38.9–43.1]	42.1 [38.4–43.8]	**0.014**
Hemoglobin, median [IQR]	14.8 [14.2–15.1]	13.6 [12.9–14.4]	14 [12.8–14.7]	**0.003**
ESR, median [IQR]	7 [5–10]	12 [6.8–19.3]	9 [5.8–14.8]	**0.028**
Platelets, median [IQR]	277200 [241800–318450]	298950 [255975–378075]	312950 [279150–325775]	0.254
Leukocytes, median [IQR]	5900 [5075–6350]	6800 [5575–7675]	5600 [4600–6300]	0.120
Lymphocytes, median [IQR]	1900 [1750–2125]	1650 [1375–2000]	1500 [1175–1900]	**0.018**
Monocytes, median [IQR]	400 [300–500]	500 [400–525]	400 [300–500]	0.179
Neutrophils, median [IQR]	3300 [2750–3825]	4400 [3300–4950]	3700 [3075–4200]	**0.013**
CRP, median [IQR]	0.26 [0.09–0.39]	0.28 [0.12–1.112]	0.31 [0.17–0.7]	0.296
C3, median [IQR]			98 [ 84.2–110.1]	
C4, median [IQR]			20.9 [13–24.2]	

### Exploring the plasma cytokine levels in RA and SLE patients

**Fig. 1:**
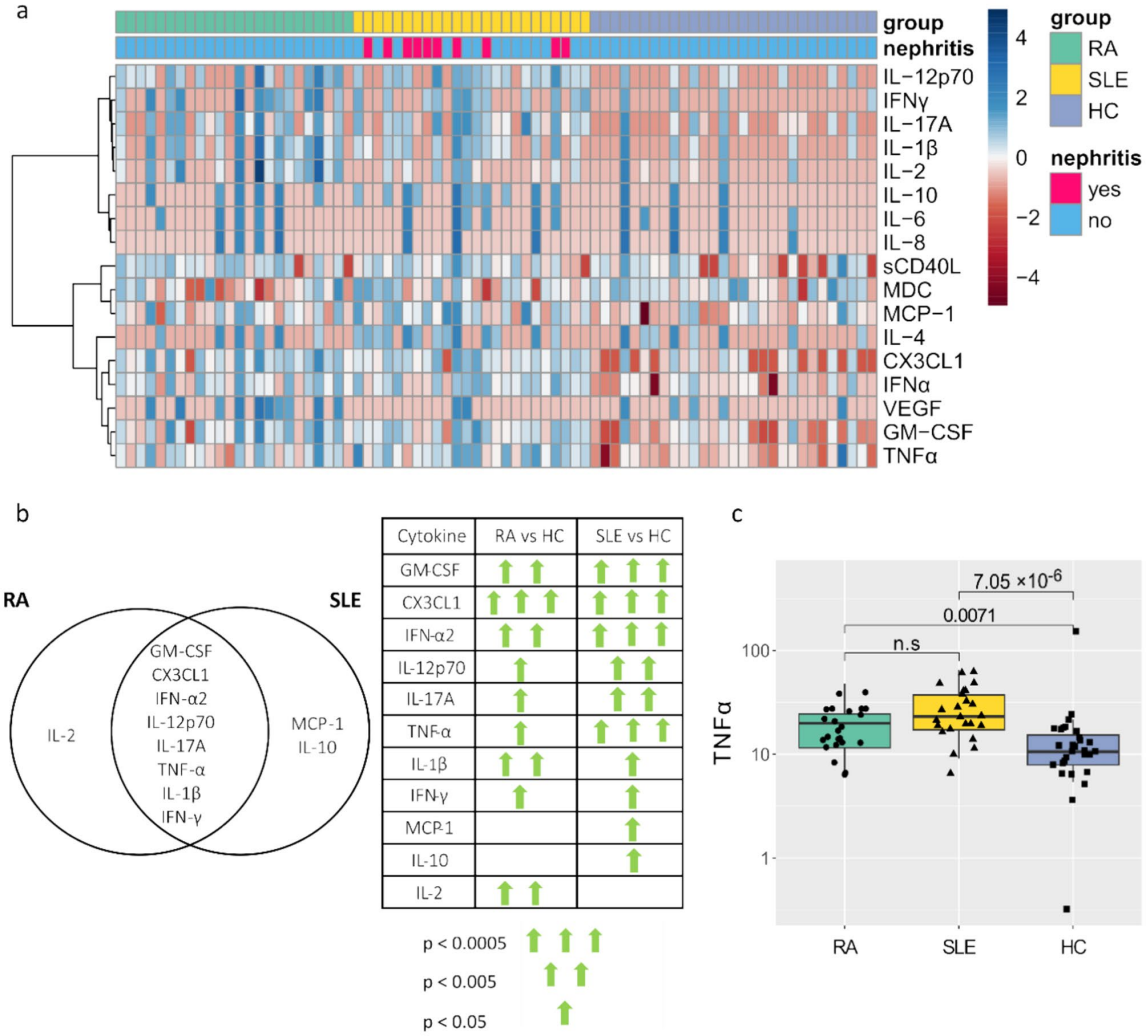
Plasma cytokine levels in Colombian RA and SLE patients. (**a**) Heatmap showing the cytokine levels and cytokine clusters. (**b**) Left: The Venn diagram illustrates the statistically significant cytokines in RA and SLE compared to HC. Right: The significance levels obtained by Dunn’s test for multiple comparisons of the cytokine alterations in RA and SLE compared to HCs are presented. (**c**) The levels of TNF-α (in pg/mL) in RA, SLE patients, and HCs. The numbers on top of the horizontal bars correspond to the *p*-values of Dunn’s test for multiple comparisons.

We performed the clustering analysis to visualize which cytokine levels have a similar variation among the RA, SLE patients, and HCs. The cytokine cluster composed of IL-12p70, IFN- γ, IL-17A, IL-1β, IL-2, IL-10, IL-6, and IL-8 is characterized because they show low levels in some of the RA and SLE patients, and therefore the levels in these patients resemble those of most of the HCs. On the other hand, in the other cytokine cluster, we found that sCD40L, MDC, MCP-1, IL-4, CX3CL1, IFN-α2, VEGF, GM-CSF and TNF-α were more consistently increased in most of the RA and/or SLE patients compared to the HCs (Fig. 1a).

The left panel of Fig. 1b shows the eleven cytokines significantly increased in RA and/or SLE. GM-CSF, CX3CL1, IFN-α2, IL-12p70, IL-17A, TNF-α, IL-1β, and IFN- γ were significantly increased in both RA and SLE, MCP-1 and IL-10 were significantly increased exclusively in SLE and IL-2 in RA. VEGF was the only cytokine that was significantly increased in RA vs SLE (Table S2). The cytokines median values, range, and the *p*-values of the multiple comparisons are shown in supplementary Table S2. The right panel of Fig. 1b summarizes to which extent the *p*-values of Dunn´s test resulted statistically significant for RA vs HC and SLE vs HCs. We focused on TNF-α for further analysis because it displayed the greatest statistical significance among all the cytokines evaluated in the comparison of SLE vs HC, but it also showed a significant *p*-value for RA vs HC (Fig. 1c).

### TNF-α and CX3CL1 levels correlated with HDL

We evaluated the correlation of the cytokine levels with blood and clinical parameters through the Spearman correlation test. Despite observing many statistically significant correlations, they were characterized by correlation coefficients (rho) with magnitudes less than or equal to + /- 0.4. This indicates that the strength of these correlations tended to be relatively weak. CX3CL1, IL-12p70, and GM-CSF have the greatest number of significant correlations with blood cell counts in RA, while MCP-1 and MDC have the most significant correlations with inflammatory markers and disease activity in SLE (Table 2). In RA, significant correlations include CX3CL1 with HDL (rho = -0.42, *p* = 0.0403) and GM-CSF with platelets (rho = 0.5, *p* = 0.0123). In SLE patients, significant correlations were also observed, such as TNFα with HDL (rho = -0.48, *p* = 0.0172) and MCP-1 with ESR (rho = 0.5, *p* = 0.0114) (Table 2).

**Table 2:** Significant Correlations between Cytokines and blood/clinical parameters in RA and SLE Patients.

RA				RA				SLE			
Var 1	Var 2	rho	*p*-value	Var 1	Var 2	rho	*p*-value	Var 1	Var 2	rho	*p*-value
CX3CL1	HDL	-0.42	0.04	TNFα	HbA1c	-0.43	0.04	MCP-1	HbA1c	0.42	0.04
	Lymphocytes	0.48	0.02		CRP	0.49	0.02		ESR	0.51	0.01
	Leukocytes	0.49	0.01	sCD40L	Hematocrit	0.42	0.04		Platelets	0.41	0.05
	Platelets	0.46	0.03		DAS28 ESR score	0.49	0.01		CRP	0.65	0.05
	Neutrophils	0.48	0.02	IL-6	Disease duration	-0.42	0.05	MDC	LDL	-0.50	0.01
IL-12p70	HDL	-0.45	0.03		DAS28 ESR score	0.62	0.00		Total cholesterol	-0.48	0.02
	Neutrophils	0.43	0.04	IL-8	Disease duration	-0.53	0.01		Monocytes	0.52	0.01
	CRP	0.47	0.02		DAS28 ESR score	0.57	0.00	TNFα	HDL	-0.48	0.02
	Monocytes	0.44	0.03	IL-1β	Leukocytes	0.42	0.04		ESR	0.45	0.03
	Lymphocytes	0.54	0.01	IFNγ	Monocytes	0.45	0.03	IL-8	HDL	-0.41	0.04
	Leukocytes	0.52	0.01	IL-17A	CRP	0.41	0.05		Lymphocytes	-0.41	0.05
GM-CSF	Leukocytes	0.54	0.01	IL-2	CRP	0.41	0.04	sCD40L	Hematocrit	-0.42	0.04
	Platelets	0.50	0.01	VEGF	CRP	0.42	0.04	IFNγ	Platelets	0.43	0.04
	Lymphocytes	0.54	0.01	IL-10	DAS28 ESR score	0.43	0.04				
	Neutrophils	0.49	0.02	MDC	DAS28 ESR score	-0.45	0.03	VEGF	Platelets	0.44	0.03
IFNα	Lymphocytes	0.42	0.04								
	Platelets	0.47	0.02	IL-4	DAS28 ESR score	0.59	0.003	IL-4	Disease duration	0.43	0.03
	Disease duration	-0.46	0.03								

**Fig. 2:**
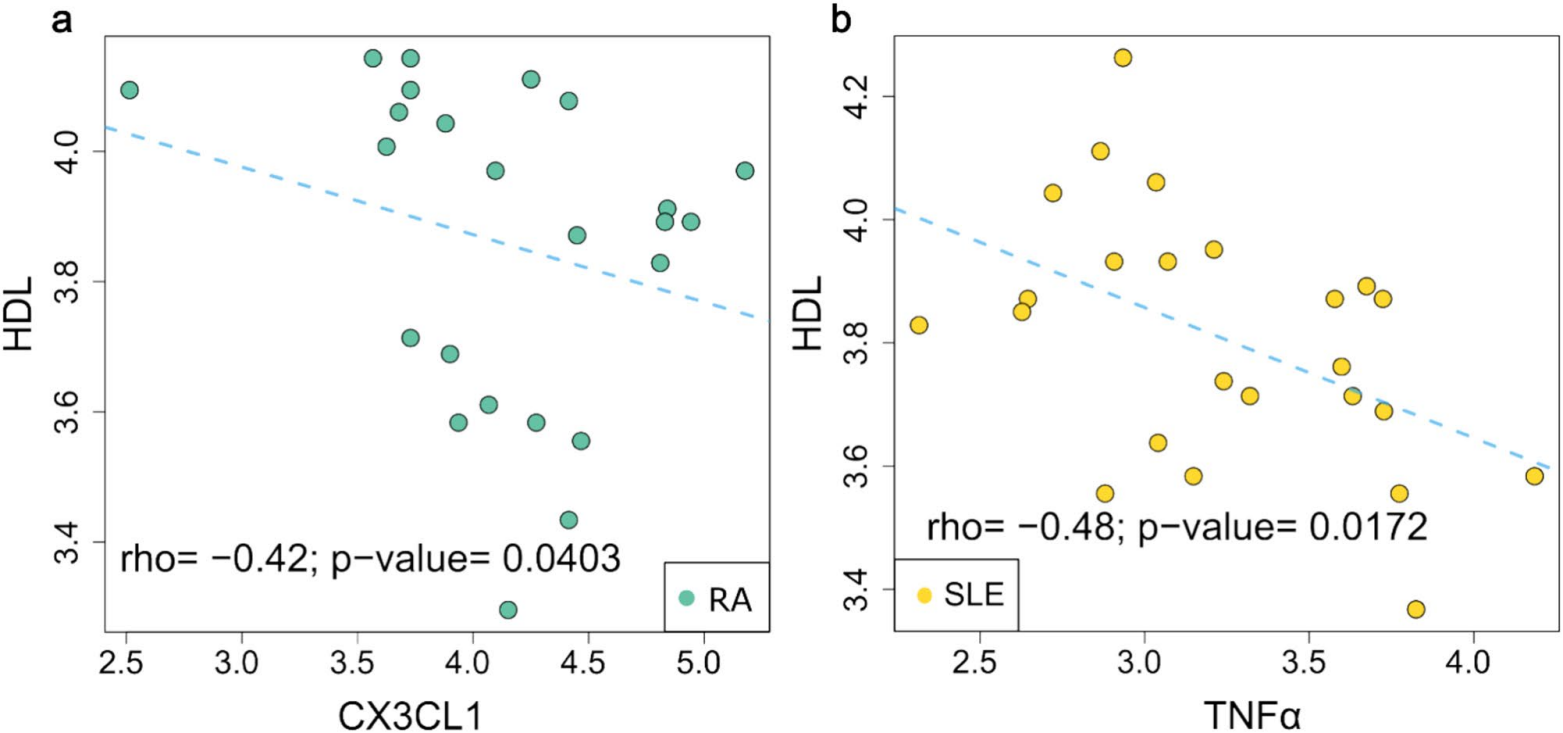
Correlation between CX3CL1 and TNF-α levels with HDL in RA and SLE patients. Scatter plot depicting the relationship between CX3CL1 and HDL levels in RA patients (**a**) and TNF-α and HDL levels in SLE patients (**b**). The dashed lines represent the linear regression analysis with corresponding rho values and *p*-values.

The negative correlation between CX3CL1 and TNF-α levels and HDL in RA and SLE respectively, suggests a potential association between these inflammatory markers and lipid profile parameters in autoimmune diseases (Fig. 2). CX3CL1, a molecule expressed on vascular endothelial cells, acts as a chemoattractant for immune cells such as monocytes, natural killer cells, and T cells. Its expression is upregulated by pro-inflammatory cytokines like TNF-α, IL-1, and IFN-γ^25^. TNF-α is a cytokine produced mainly by macrophages, T lymphocytes, and natural killer cells, and it is known for not being exclusively pro-inflammatory or anti-inflammatory^26^. TNF-α is a key inflammatory cytokine that orchestrates the body’s immune response to infection and sepsis, while also influencing a broad spectrum of biological processes encompassing inflammation, cell survival, and death^27^. TNF-α is a well-established mediator of acute phase protein synthesis in hepatocytes, including CRP and α1-acid glycoprotein (α1AG). In the context of SLE, TNF-α exhibits moderate effects in the production of acute-phase proteins^28^.

### IL-4 cytokine network and its association with SLE

**Fig. 3:**
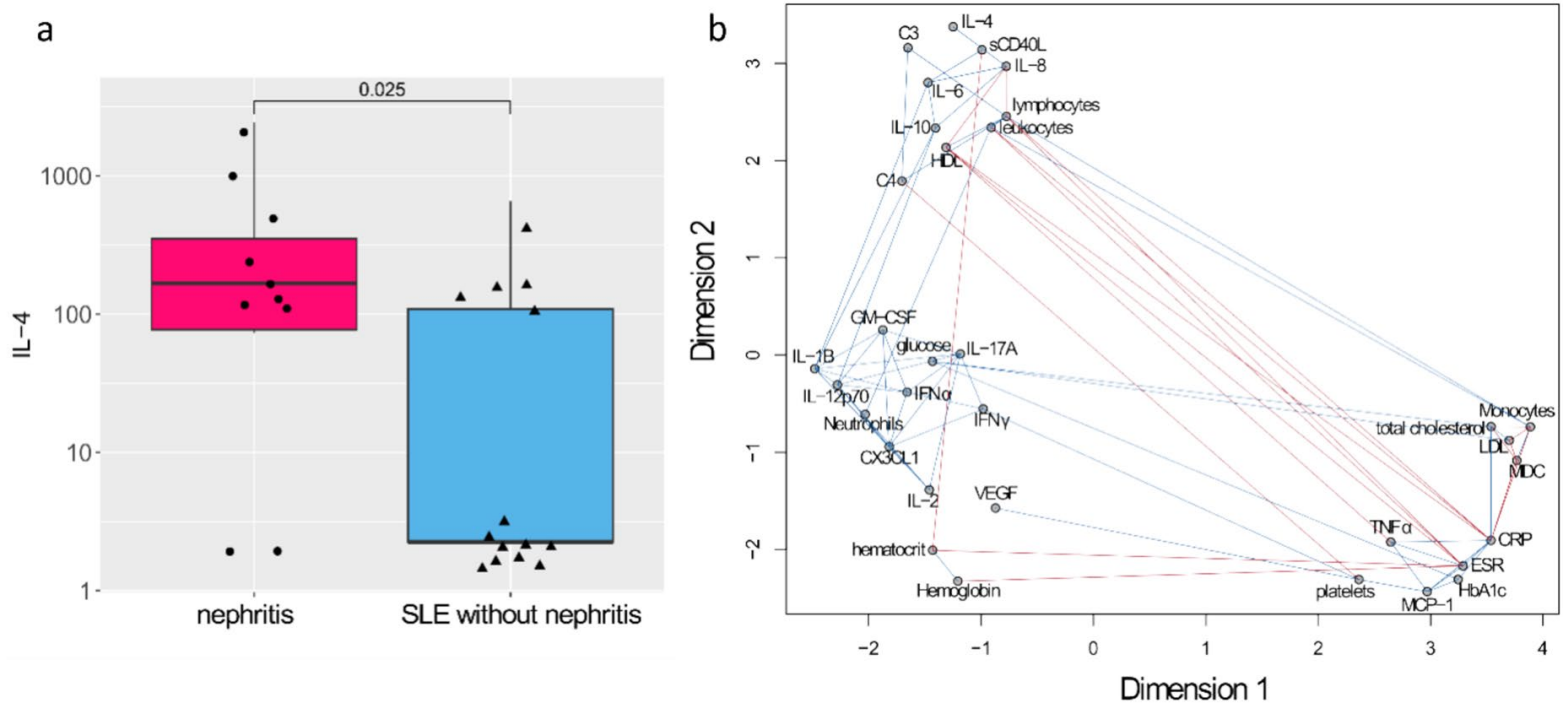
IL-4 cytokine network and its association with SLE. (**a**) The levels of IL-4 (in pg/mL) in SLE patients with and without nephritis. The number on top of the horizontal bar corresponds to the *p*-value of the Wilcoxon rank-sum test. (**b**) Network analysis of the correlations between cytokines and blood parameters in SLE. The positive correlations are shown as blue lines and the negatives as red lines.

IL-4 levels were higher in SLE compared to HCs and RA but there was no statistical significance (Table S2). However, the comparison of the cytokine levels between SLE patients with and without nephritis was also performed, and it was found that IL-4 was significantly elevated in the patients with lupus nephritis (Fig. 3a). IL-4 is a multifunctional cytokine with roles like B cell activation, Th2 promoting properties, T cell suppression, and contributing to target organ damage^29^. We wanted to assess in SLE the relationship between IL-4 and the other cytokines as well as the blood parameters. To this end, we performed a network analysis of the correlations between these variables, and we found that IL-4 forms a correlation cluster with other cytokines (i.e., sCD40L, IL-8, IL-6, and IL-10), but also with blood parameters (i.e., C3, C4, lymphocytes, leukocytes, and HDL) (Fig. 3b). It is important to highlight the central position of IL-6 in this cytokine cluster.

## Discussion

This study allowed us to characterize the cytokine profile of a Latin American population of RA and SLE patients. The statistically significant increase of GM-CSF, CX3CL1, IFN-α2, IL-12p70, IL-17A, IL-1β, IFN-γ, and TNF-α in both RA and SLE compared to HCs is explained by the autoimmune tautology since they share genetic and environmental risk factors but also pathophysiological mechanisms^30^. Some cytokines with mainly proinflammatory functions such as TNF-α, IL-6, IL-17, MCP-1, IFN-α, IFN-γ, and GM-CSF are elevated in the plasma of patients with RA^11,31–34^. Our RA patients had significantly increased levels of IL-2, which is a pleiotropic cytokine important for the proliferation of effector T lymphocytes^35^. In SLE, there have been found elevated cytokines like IFN-α, IL-6, IL-12, sCD40L, IL-10, TNF-α, IL-8, IL-4, and MCP-1^11,31,36–42^. We found that MCP-1 and IL-10 were significantly increased exclusively in the SLE patients as previously described. The role of MCP-1 is to recruit monocytes and macrophages to sites of inflammation^43^ and IL-10 is involved in B-cell differentiation and autoantibody production^44^. It is worth noting that these previous results were obtained from RA and SLE patients with different ethnicities but not Latin American. Therefore, the results of this study can be applied to understand better cytokine biomarkers and targets within the Colombia admixed population.

CX3CL1, produced by synovial tissue cells including macrophages, fibroblast-like synoviocytes, endothelial cells, and dendritic cells, is highly expressed in RA synovial fluid and is thought to contribute to the adhesion, migration, and cytokine production of inflammatory cells within the synovium^25^. The critical role of CX3CL1 in joint destruction is further supported by studies demonstrating that anti-CX3CL1 antibody treatment suppresses osteoclast precursor migration, reduces osteoclast formation, and inhibits bone erosion in a collagen-induced arthritis model. These findings suggest that the CX3CL1/CX3CR1 pathway is crucial in joint destruction by regulating osteoclast precursors migration^45^. Given the elevated CX3CL1 levels in RA patients and its established role in disease progression, targeting this chemokine presents a promising therapeutic strategy.

TNF-α is one of the most studied and clinically targeted cytokines in the treatment of autoimmune diseases and its blockade has been successful in RA^46^. TNF-α is central to RA pathophysiology because it mediates the activation and effector functions of leukocytes, endothelial cells, and osteoclasts^47^, therefore we expected to find this cytokine significantly elevated in our cohort of RA patients. On the other hand, conflicting results have been found regarding the role of TNF-α in SLE because there is evidence suggesting that it is upregulated in SLE patients but attempts to block this cytokine in the context of SLE have not been a successful treatment strategy, and has even resulted in exacerbations of the disease^48^. However, the data shows that TNF-α inhibitors can be used during short periods for the treatment of lupus nephritis after an induction regimen of four infusions whose effect lasted for many years, so there is a real chance TNF-α blockade can be helpful for subsets of SLE patients with refractory disease^49^. Further research is needed to understand better the effects of TNF-α antagonists in SLE, particularly in the context of personalized medicine^44^. Finding in our SLE patients greater TNF-α levels than in RA patients was somewhat surprising given that this cytokine has a more important role in RA than in SLE. Nevertheless, the possibility of targeting TNF-α in Colombian patients should be explored considering the current findings of the elevated levels of this cytokine in Colombian SLE patients but also aiming to inquire into the efficacy it may have in specific subsets of these SLE patients.

The high statistical significance of the negative correlation between CX3CL1 and TNF-α with HDL in RA and SLE respectively, has special relevance for these patients since they have an increased cardiovascular disease (CVD) risk because of the coexistence of dyslipidemia and chronic inflammation^50^. HDL has properties that are protective against CVD risk, being the most recognized their ability to promote cholesterol efflux from cells in the artery wall, but it also inhibits the oxidation of LDL reducing their atherogenicity^51^. HDL acts as an anti-inflammatory by inhibiting the hyperactivation of immune cells through cholesterol removal from the cell membrane, thus limiting the formation of lipid raft domains that serve as platforms where immune cell receptors are organized^50^. Reduced levels of HDL have been previously described in RA and SLE, but they also display an altered composition and function that makes them proinflammatory HDL (pro-HDL) that contributes to the development of atherosclerosis^52^. The pro-HDL has been found in 44.7% of SLE patients but it has also been reported in 20.1% of RA patients^53^. Therefore, these pro-HDL are expected to be present as well in our RA and SLE patients.

The CX3CL1-CX3CR1 pathway plays a crucial role in atherosclerosis, contributing to leukocyte recruitment and vascular inflammation**.** Genetic variations in the CX3CR1 gene have been linked to an increased risk of coronary artery disease, further emphasizing the importance of this pathway in cardiovascular health^54^. TNF-α contributes to metabolic dysfunction by inducing insulin resistance, increasing hepatic glucose production, and promoting lipid accumulation through enhanced hepatic lipid synthesis and adipose lipolysis while inhibiting lipid clearance^55^. Furthermore, the regulation of ATP-binding cassette transporter A1 (ABCA1) and HDL cholesterol efflux by TNF-α was investigated in the human intestinal cell line Caco-2. ABCA1 transporter is crucial for initiating HDL formation by facilitating the efflux of cholesterol and phospholipids to apolipoprotein A1. TNF-α suppresses ABCA1 expression and function, leading to decreased HDL cholesterol efflux. This inflammatory cytokine inhibits both ABCA1 transcription and protein stability, requiring an active NF-κB pathway for maximal effect^56^. The CX3CL1-CX3CR1 pathway and TNF-α are key drivers of inflammation and atherogenesis, contributing significantly to the heightened CVD risk in RA and SLE patients. Targeting these pathways presents a promising avenue for developing novel therapeutic strategies to combat these complex diseases and the associated CVD risk**.**

The finding that IL-4 is significantly increased in SLE patients with nephritis is tightly linked to the end-organ effects of this cytokine. In a lupus mice model that develops progressive renal disease it was found a high level of IL-4, so anti-IL-4 treatment reduced proteinuria and prolonged mice survival^29^. The roles of cytokines within a complex regulatory network are related to specific immunological processes that promote autoimmunity, chronic inflammation, and tissue destruction. Elucidating such networks can present novel opportunities for controlling the disease and for the induction of disease remission^57^. In this study we discovered in SLE patients a cytokine network whose central node is IL-6, but where we also found IL-8, sCD40L, IL-4, and IL-10. The fact that IL-6, sCD40L, IL-4, and IL-10 are tightly linked in the same cluster is reasonable considering that these cytokines share an important role in B cell responses like promoting B cell proliferation, isotype switching, sustaining the production of autoantibodies and rescuing B cells from apoptosis^44^. IL-8 has an indirect role in promoting B cell responses since it induces the neutrophil extracellular trap formation which provides the nuclear antigens for the continuous production of autoantibodies^44^.

We excluded patients with major organ involvement to ensure a more homogeneous group of SLE patients, acknowledging the inherent heterogeneity of SLE. The RA and SLE patients included in this study had a minimum of two years of disease duration so we expected them to be taking medications that may have affected their cytokine profile. It is also important to highlight that once these patients are diagnosed, they will be prescribed drug treatments. Therefore, the most common scenario in our country is to find patients receiving drug treatments and our study reflects this reality. However, we proved that the patients are not clustered according to the drug treatments in the multivariate analysis (Fig. S1). Despite the study's sample size, which can reduce statistical power, we described interesting results. We acknowledge that plasma cytokine profiles may not always accurately reflect cytokine levels within tissues. This limitation should be considered when interpreting our findings.

## Conclusion

The cytokines elevated in common in RA and SLE are evidence of the shared inflammatory signature in these autoimmune diseases, as expected by the autoimmune tautology. This study focused on TNF-α, which had been considered important only for RA physiopathology, but that we found with higher statistical significance in SLE vs HC despite the controversial results in other studies. Interestingly, the negative correlation of CX3CL1 and TNF-α with HDL might reflect the relevance of these cytokines in the CVD risk seen for RA and SLE patients. Therefore, we highlighted the possibility of blocking these cytokines to ameliorate these complex diseases and their associated CVD risk**.** We also discovered the increase of IL-4 in SLE patients with nephritis and how it forms a correlation cluster with IL-6, IL-10, sCD40L, and IL-8, which might be related to the fact that B cell responses are a keystone in the development of SLE nephritis. This study has revealed the importance of CX3CL1, TNF-α, and IL-4 as candidates to be studied as potential drug targets for Colombian SLE patients.

## Supplementary Information

Supplementary Figure 1.

Supplementary Tables.

## Data Availability

Data is available upon request to the corresponding author.
