# Supplementary material for: Cytokine profiles and their correlation with clinical and blood parameters in rheumatoid arthritis and systemic lupus erythematosus
Source: Sci Rep. 2024 Oct 8;14:23475. doi: 10.1038/s41598-024-72564-z (PMC11461704; doi:10.1038/s41598-024-72564-z)
Supplement: Supplementary file 1 — Supplementary Figure 1. [file 41598_2024_72564_MOESM1_ESM.pdf]

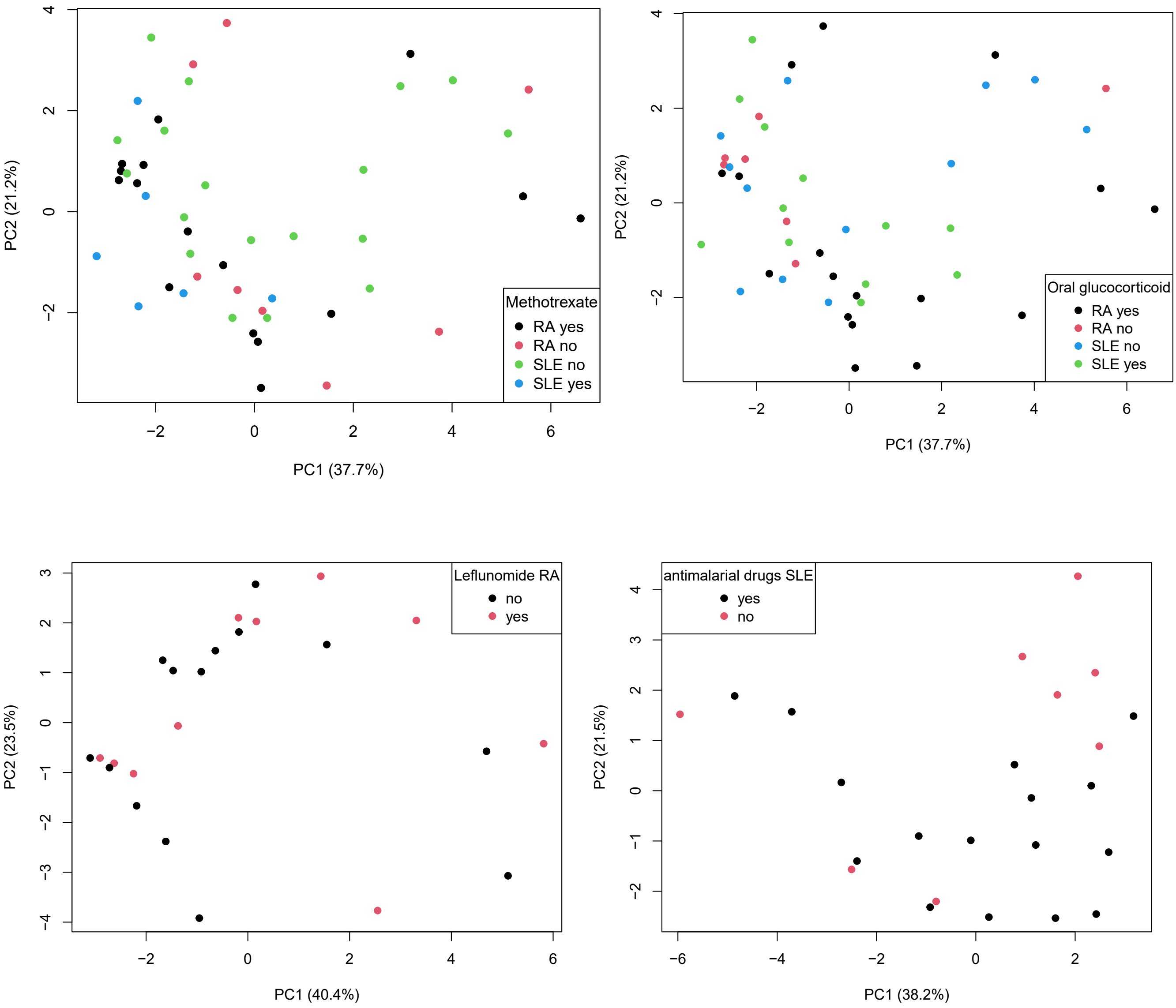

Figure S1. Principal component analysis showing the two-dimensional distribution of the RA and SLE patients according to the levels of the 17 cytokines measured. The different plots show the effect of the drug treatments in the two-dimensional distribution of the patients.
